# Cerebellar abiotrophy in an Icelandic horse

**DOI:** 10.1186/s13028-022-00651-0

**Published:** 2022-11-26

**Authors:** Sanni Hansen, Emil Olsen, Marie Raundal, Jørgen Steen Agerholm

**Affiliations:** 1 Department of Veterinary Clinical Sciences, Faculty of Health and Medical Sciences, University of Copenhagen, Agrovej 8, 2630 Taastrup, Denmark; 2 University Animal Hospital, Swedish University of Agricultural Sciences (SLU), Box 7040, 75007 Uppsala, Sweden; 3 Raundal Equine Practice, Brusenvej 3, 7500 Holstebro, Denmark

**Keywords:** Abiotrophy, Ataxia, Cerebellum, Congenital, Equine, Genetic, Hereditary

## Abstract

**Background:**

Cerebellar abiotrophy (CA) is an uncommon hereditary neurodegenerative disorder affecting the cerebellar Purkinje cells. Equine CA has been reported in several breeds, but a genetic etiology has only been confirmed in the Arabian breed, where CA is caused by an autosomal recessive mutation.

Case presentation.

Clinical and histological findings consistent with CA are reported in an 8.5-month-old Icelandic filly. The filly showed a perceived sudden onset of marked head tremor, incoordination, ataxia, lack of menace response and a broad-based stance. Cerebrospinal fluid, hematological and biochemical findings were all within the normal range, ruling out several differential diagnoses. Post mortem histopathological examination revealed Purkinje cell degeneration accompanied by astrogliosis. Assessment of the filly’s pedigree revealed that its parents shared a common ancestor.

**Conclusions:**

To the authors’ knowledge, this is the first report of CA in the Icelandic breed. The identification of a common parental ancestor makes autosomal recessive inheritance of CA in this filly possible, but this would need to be confirmed by further studies. Veterinarians and breeders working with Icelandic horses should be aware of this condition and report suspected cases in order to support genetic investigation.

**Supplementary Information:**

The online version contains supplementary material available at 10.1186/s13028-022-00651-0.

## Background

Cerebellar abiotrophy (CA) is an uncommon neurodegenerative disorder of young animals characterized histologically by progressive postnatal degeneration of Purkinje cells with subsequent neuronal death [1, 2]. Purkinje cell degeneration results in non-fatal clinical signs including a lack of menace response, intention tremors, ataxia with exaggerated action of the thoracic limbs and a wide-based stance of the pelvic limbs [3]. Clinical signs often develop between the ages of 6 weeks and 4 months, and are often perceived as having a sudden onset. While CA has been reported in several domestic animal species [4], equine CA has primarily been described in the Arabian breed [2, 5], where its development has been associated with an autosomal recessively transmitted single base mutation in the *TOE1* gene [5, 6]. This mutation has been transmitted to the Trakehner, Welsh pony and Bashkir Curly horse breeds due to cross breeding [7]. Cerebellar abiotrophy has also been reported in a number of other breeds such as the New Caledonia horse [8], Oldenburg [9], American Miniature Horse [10] and the Gotland pony [11].

A genetic test has been developed and validated for the Arabian breed, while for other breeds, a genetic cause is suspected but not yet proven [3].

We report a case of CA in an Icelandic horse and propose the existence of a hitherto unrecognized inherited form of CA in that breed.

## Case presentation

An 8.5-month-old Icelandic filly was presented to the primary care veterinarian due to what the owner perceived as a sudden onset of neurological signs. The filly was perceived as being previously healthy, except for an upper respiratory tract infection and fever 3 weeks prior to presentation. The filly was born at the premises and had been with the same owner and at the same farm since birth. The initial clinical signs consisted of head tremors and hypermetria of all four limbs and stiff gait on both thoracic limbs. The filly was treated supportively with dexamethasone (0.1 mg/kg IM for 2 days; Dexadreson^®^ Vet., 2 mg/mL, Intervet International B.V., The Netherlands). However, the condition progressed to marked ataxia of all four legs and difficulty rising within a few days. Based on the history and clinical signs, a progressive cerebellar lesion was suspected. Due to a poor prognosis and the markedly progressive nature of the condition, the owner donated the filly to the University of Copenhagen, Denmark for research. The filly was deemed safe for transportation and brought to the University Large Animal Teaching Hospital for clinical and postmortem examination.

According to the owner, another offspring (male) of the dam, bred to another sire and born 4 years prior had developed neurological signs at the age of 3 months. The clinical signs of this half-sibling also occurred suddenly and were characterized by an apparent loss of orientation and anxious behavior. A detailed neurological examination was not performed at that time and the foal was euthanized within a few days, as the condition did not improve and the foal was disposed without post mortem examination.

The clinical examination was performed the day after arrival to the hospital. The filly was bright and alert, slightly anxious but with normal mentation. It was of normal body condition (score of 4/9) [12] and with rectal temperature, heart rate and respiratory rate all within normal limits. The submandibular lymph nodes were indolent and bilaterally of normal size, and the nares were with minimal serous exudate. The oral mucosa was pink with a capillary refill time of 1–2 s. Auscultation of the heart and lung revealed no abnormalities. Intestinal borborygmi was normal over all four quadrants. No lameness was observed, and the digital pulses were of normal strength on all four legs.

When fed or given treats, as well as when initiating movement and navigating through a narrow space with her head, the filly had marked intention tremors also involving the neck and body, resembling titubation.

During the neurological examination, no head tilt or head turn was observed, the medial and lateral palpebral reflexes were normal, as were the direct and indirect pupillary light reflexes. The menace response was bilaterally absent. The oculocephalic reflex (physiological nystagmus) was normal bilaterally and she had no spontaneous nystagmus or strabismus with her head in a normal position, however, there was a moderate bilateral positional ventral strabismus when the head was moved vertically. There were no signs of muscle atrophy, and activity in the fascial muscles was normal. The filly had normal sensation in the planum nasale, but a mildly exaggerated response to a touch of the face or body was noted.

During walk, tölt and trot, the filly was markedly hypermetric on all four limbs, slightly worse on the left thoracic and left pelvic limbs (see video in Additional files 1, 2 and 3). When rising from a lying position in the stall, the filly would fall towards the left, but she would be able to correct herself and get up.

The filly did not circumduct during circling and was able to back up as well as navigate low obstacles without mistakes, thus suggesting intact proprioception. When the head was elevated, there were no changes in gait. When standing and initiating movement or navigating through a narrow space, the filly had marked truncal sway and intention tremors. Normal tonus was present when the tail was pulled at stand-still, but there was less resistance than expected during walk.

The lesion was neuroanatomically localized to the cerebellum based on the bilaterally absent menace response, the left-lateralizing hypermetria, as well as intention tremors and a bilateral positional ventral strabismus.

The primary differential diagnoses for a progressive cerebellar lesion with no pain, slightly lateralizing to the left, were CA, a congenital anomaly (e.g., cerebellar hypoplasia, sub-cerebellar cyst) or a metabolic condition (e.g., neuronal ceroid lipofuscinosis). Infectious/inflammatory conditions were considered highly unlikely due to the history, but could not be ruled out completely.

Cerebrospinal fluid was obtained immediately after euthanasia via the cerebello-medullary cistern. The aspirate was clear and colorless and cytology findings were within normal range with only a few mononuclear cells and occasional erythrocytes present.

An EDTA blood sample was analyzed for the single base polymorphism in the *TOE1* gene associated with CA in Arabian horses [13], but found homozygous for the wildtype-allele (LABOKLIN, Bad Kissingen, Germany).

Hematological and biochemical parameters were within normal ranges, except for a slightly increased level of fibrinogen (4.14 g/L; ref. 1–4 g/L; Additional file 4), which was considered clinically non-significant. A nasal swab sample taken by the primary care veterinarian prior to admission to the university hospital was positive for *Streptococcus equi* subsp. *zooepidemicus* and Equid herpesvirus type 5.

The filly was euthanized by an overdose of barbiturate intravenously and necropsied. No gross lesions were found, with the exception of localized subcutaneous hemorrhage and edema above the right sacral tuber, several chronic gastric ulcerations in the non-glandular portion and some *Gasterophilus* larvae attached to the epithelium. The central nervous system (CNS) was unremarkable and with a total brain weight of 469.5 g and a cerebellar weight of 33.5 g (7.1%). The brain was fixed *in toto* by immersion in 10% neutral buffered formalin, as was the spinal cord, peripheral nerves, skeletal musculature and specimens of visceral organs. After fixation, the brain was embedded in agar, sectioned in 4 mm parallel transverse slices, visually inspected for gross lesions, and then subdivided into smaller pieces. Gross lesions were not observed. Selected regions of the CNS, spinal cord and other tissues were processed for histopathology, paraffin embedded, sectioned at 4 μm and stained with hematoxylin and eosin. Sections of the cerebellum were also stained with Luxol fast blue and immunohistochemically analyzed for expression of glial fibrillary acidic protein (Z0334 Agilent Technologies Denmark ApS**,** Glostrup, Denmark) using the UltraVision HRP system (Fisher Scientific, Fremont, CA, USA).

**Fig. 1:**
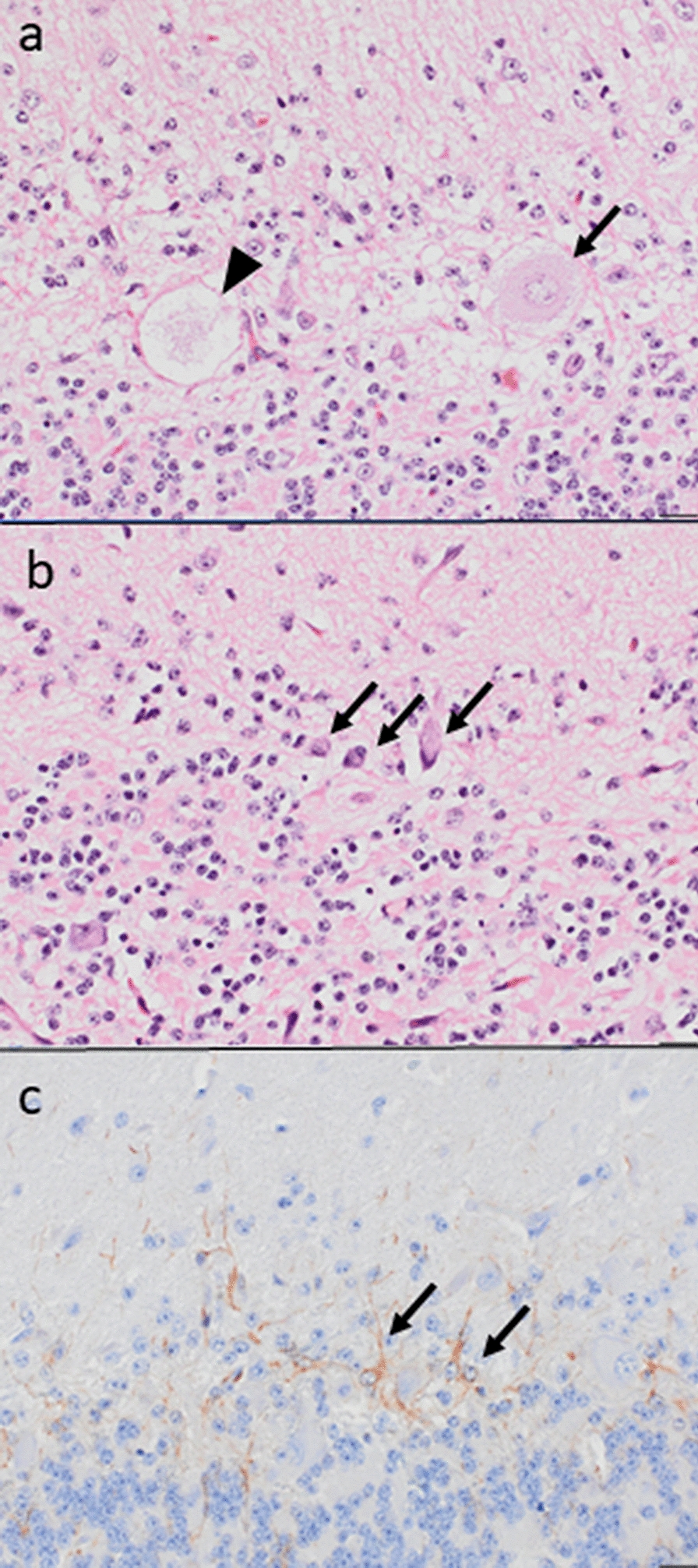
Photomicrographs of cerebellar lesions. **a** Cytoplasmatic remnants of a Purkinje cell are seen within an empty basket (*arrowhead*), while another degenerated Purkinje cell has pale cytoplasm, a large pale nucleus and poorly defined borders (*arrow*); **b** Shrunken Purkinje cells with sparse amounts of cytoplasm (*arrows*); **c** Immunohistochemical staining for glial fibrillary acidic protein showing astrogliosis in the Purkinje cell layer (*arrows*). Bar = 20 µm; a-b: hematoxylin and eosin; c: UltraVision HRP

Histopathological examination revealed an extensive and diffuse loss of Purkinje cells. Wide parts of the Purkinje cell layer lacked Purkinje cells and appeared as a spongy zone, while scattered normal-appearing Purkinje cells were present in other parts alongside degenerated Purkinje cells. These appeared either as large cells with pale cytoplasm, large pale nuclei and poorly defined borders, sometimes only as cytoplasmic remnants, or as shrunken hyperchromatic cells with sparse amounts of cytoplasm (Fig. 1a, b). Shrunken Purkinje cells were also scattered in the profound molecular layer. Depletion of granule cells was mild and inconsistent. Immunohistochemistry revealed astrogliosis in the Purkinje cell and profound molecular layers (Fig. 1c). Other parts of the brain, the spinal cord, peripheral nerves, skeletal muscle and other tissues were unremarkable, except for a chronic granulomatous steatitis in the sacral tuber area.

**Fig. 2:**
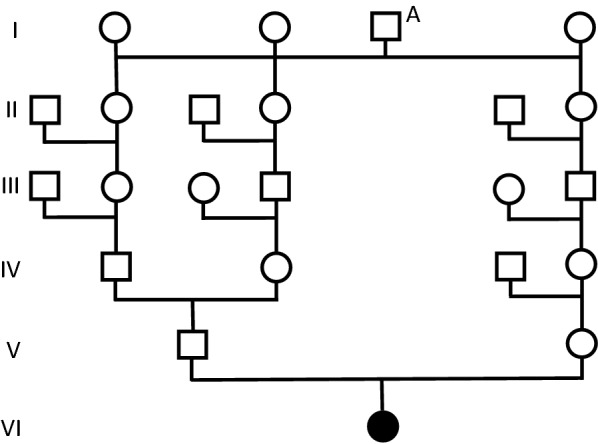
Genealogic diagram showing the familial relationship between the parents of the filly affected by cerebellar abiotrophy. Only the most relevant individuals are shown. The common ancestor is marked by A. ●: Affected filly; ○: Mare; □: Stallion

Examination of six-generation pedigree data revealed that the parents of the CA-affected filly were genetically related though a common ancestor. The ancestor (A) was a stallion born in Iceland in 1968. He was related to the filly’s dam through one of his daughters and to the filly’s father through two of his daughters (Fig. 2). Pedigree analysis of the half-sibling that developed neurological signs 4 years previously revealed a genetic relationship to the sire of stallion A.

## Discussion and conclusions

Based on an absent menace response with normal palpebral reflexes and the left-lateralizing hypermetria, as well as intention tremors during eating and a positional ventral strabismus, a lesion neuroanatomically localized to the cerebellum was suspected, with CA as the most likely cause. Neuropathological examination revealed selective degeneration and loss of Purkinje cells in the cerebellum and thus confirmed the diagnosis of CA.

Cerebellar abiotrophy has, to the authors’ knowledge, not previously been reported in Icelandic horses. In other breeds such as the Gotland pony, CA has occurred in a familial pattern consistent with the transmission of an autosomal recessive allele [11], and equine CA is generally regarded a disease with a genetic etiology [3], although other causes cannot be completely excluded. In Arabian horses, a single base mutation in the *TOE1* gene associated with development of CA has been identified, thus confirming the genetic cause in that breed [5, 6]. Pedigree analysis of the current case revealed that a CA-associated recessive allele present in stallion A could have been transmitted to the filly through its parents, thus causing homozygosity and subsequent development of CA. Although stallion A may have been in the pedigree of both parents by coincidence, or a recessive allele could have been introduced into the pedigree of the filly by other individuals, the presence of inbreeding loops means that a genetic cause of CA in the filly is likely. This calls for an international awareness among breeders of Icelandic horses. Although stallion A was born in Iceland, the presence of a CA-affected filly in Denmark shows that the condition may have spread to subpopulations of Icelandic horses outside of Iceland.

While CA is often clinically inapparent in newborn foals, clinical signs can develop within 6 weeks to 6 months after birth. A small number of cases have presented with congenital clinical signs [14] and further cases of adult-onset CA have also been reported [15]. The current filly presented with clinical signs at the age of 8.5 months, i.e., slightly older than the usual presentation of equine CA. Most knowledge on equine CA originates from studies in the Arabian breed, but CA in the Oldenburg breed has also been reported with progressive and fatal clinical signs [9].

The filly presented with a history of an apparently sudden onset of clinical signs. While sudden onset has previously been reported [14], mild intention tremors may have gone unnoticed by the owners as the filly was stabled in a loose-stable environment with many horses, and it is possible that clinical signs may not have been observed before they became quite severe.

The hematology and biochemistry findings were within normal ranges, as previously reported [14]. Analysis of cerebrospinal fluid of CA-affected horses has shown an increase in protein concentration as the only abnormality [14]. In this case, protein was not measured, but other parameters were within the normal ranges.

At necropsy, the total weight of the filly’s brain was 469.5 g and the cerebellar weight was 33.5 g, representing 7.1% of the total brain, which is lower than normal [16] and in accordance with the CA diagnosis.

The presence of a common parental ancestor (stallion A) means that it is possible that CA was caused by the transmission of an autosomal recessive allele through both parents. However, as a common parental ancestor may appear in the pedigree by coincidence, the presence of a recessive allele causing CA in Icelandic horses is currently hypothetical. If present, the molecular basis differs from that found in Arabian horses as the filly was homozygous for the Arabian-horse-specific wildtype allele. The Icelandic horse is unique in its genetic pool, as it has been isolated in Iceland, so the likelihood of CA in this breed being caused by a mutation identified in another breed is low [17]. If present, the mutation is therefore likely to be unique to the Icelandic breed. It is important for the Icelandic horse breed that any future cases are identified and verified by histopathology, thus enabling sampling of tissues for genetic analysis and development of a genotyping test.

Icelandic horses that develop clinical signs indicating CA such as an absent or inconsistent menace response, intention tremors in combination with dysmetria and a wide-based stance should therefore be investigated thoroughly and referred for postmortem and genetic investigation. A general awareness among the Icelandic breeding industry should be supported, so that foals with clinical signs indicating CA can be examined.

## Supplementary Information

**Additional file 1**: The filly led by hand in walk shows marked hypermetric gait on all four limbs. MOV- file.

**Additional file 2**: The filly led by hand in tölt and trot shows marked hypermetric gait on all four limbs, slightly more pronounced on the left thoracic and left pelvic limbs. MOV- file.

**Additional file 3**: The filly encouraged to trot in the area shows marked hypermetric gait. MOV- file.

**Additional file 4**: Overview of biochemical and hematological analyses and examination for respiratory pathogens.

## Data Availability

The datasets used and/or analyzed during the current study are available from the corresponding author on reasonable request. Data have not been published previously.
