# Supplementary material for: Cerebellar abiotrophy in an Icelandic horse
Source: Acta Vet Scand. 2022 Nov 26;64:31. doi: 10.1186/s13028-022-00651-0 (PMC9701424; doi:10.1186/s13028-022-00651-0)
Supplement: Supplementary file 4 — Additional file 4: Overview of biochemical and hematological analyses and examination for respiratory pathogens. [file 13028_2022_651_MOESM4_ESM.docx]

**Additional file 4.** Overview of biochemical and hematological analyses and examination for respiratory pathogens

**Biochemical analyses**

| **Parameter** | **Case** | **Reference interval^1^** |
| --- | --- | --- |
| Alkaline phosphatase (U/L) | 175 | <450 |
| Gamma-glutamyl transferase (U/L) | 5 | 4-19 |
| Bilirubin total (µmol/L) | 41 | <52 |
| Aspartate aminotransferase (U/L) | 313 | 228-366 |
| Creatine kinase (U/L) | 197 | <348 |
| Total protein (g/L) | 61.53 | 57-74 |
| Albumin (g/L) | 33.05 | 28-40 |
| Carbamide (mmol/L) | 5.7 | 3.3 - 8 |
| Creatinine (µmol/L) | 94 | 30-130 |
| Calcium (mmol/L) | 2.78 | 2.53-3.25 |
| Magnesium (mmol/L) | 0.73 | 0.66-0.95 |
| Iron (µmol/L) | 13.3 | 13.1 - 43 |
| Serum amyloid A (mg/L) | 3 | <30 |

^1^ Alkaline phosphatase analyzed at LABOKLIN, Bad Kissingen, Germany. All other were

analyses performed at the Department of Veterinary Clinical Sciences, University of Copenhagen.

**Hematological analyses**

| **Parameter** | **Case** | **Reference interval^1^** |
| --- | --- | --- |
| Erythrocytes (T/L) | 8.86 | 6.6-11.4 |
| Hematocrit (L/L) | 0.35 | 0.32-0.5 |
| Hemoglobin (g/L) | 7.6 | 6.82-11.06 |
| Leukocytes (G/L) | 10.74 | 5.45-12.65 |
| Neutrophils (%) | 50.6 | 28-82.8 |
| Lymphocytes (%) | 44.6 | 19.8-58.9 |
| Monocytes (%) | 1.5 | 1.4-10.5 |
| Eosinophils (%) | 2 | <8.7 |
| Basophiles (%) | 1 | <2 |
| Thrombocytes (G/L) | 137 | 100-350 |
| Neutrophils (G/L) | 5.44 | 2.26-7.22 |
| Lymphocytes (G/L) | 4.79 | 1.26-5.74 |
| Monocytes (G/L) | 1.16 | <1 |
| Eosinophils (G/L) | 0.21 | <1 |
| Basophils (G/L) | 0.11 | <0.29 |

^1^ Analyzed at the Department of Veterinary Clinical Sciences, University of Copenhagen.

**PCR analysis of a nasal swab for respiratory pathogens^1^**

| **Pathogen** | **Result** |
| --- | --- |
| *Streptococus equi* subsp. *zooepidemicus* | Positive |
| *Streptococus equi* subsp. *equi* | Negative |
| Influenza A virus | Negative |
| Equid herpesvirus type 1 | Negative |
| Equid herpesvirus type 4 | Negative |
| Equid herpesvirus type 5 | Positive |

^1^ Analyzed at LABOKLIN, Bad Kissingen, Germany
